# Health Insurance Enrollment Among US Veterans, 2010-2021

**DOI:** 10.1001/jamanetworkopen.2024.30205

**Published:** 2024-08-01

**Authors:** Todd H. Wagner, Anna Schmidt, Forest Belli, Marion Aouad, Elizabeth Gehlert, Malav Desai, Laura Graham, Liam Rose

**Affiliations:** VA Health Economics Resource Center, VA Palo Alto, Menlo Park, California (Wagner, Schmidt, Belli, Gehlert, Desai, Graham, Rose); Stanford Surgery, Policy, Improvement, Research and Education Center, Palo Alto, California (Wagner, Belli, Desai, Graham, Rose); Department of Economics, University of California, Irvine (Aouad)

## Abstract

**IMPORTANCE:**

Department of Veterans Affairs (VA) health care spending has increased in the past decade, in part due to legislative changes that expanded access to VA-purchased care.

**OBJECTIVE:**

To understand how insurance coverage and enrollment in VA has changed between 2010 and 2021.

**DESIGN, SETTING, AND PARTICIPANTS:**

This cross-sectional study used data from surveys conducted from 2010 to 2021. Participants were respondents across 4 national surveys who reported being a US veteran and reported on health insurance enrollment. Data were analyzed from October 2023 to June 2024.

**MAIN OUTCOMES AND MEASURES:**

Self-reported health insurance coverage, reliance on VA insurance, and self-reported health.

**RESULTS:**

Among a total of 3 644 614 survey respondents (mean [SE] age, 60 [0.04] years; 91.3% [95% CI, 91.2%-91.5%] male) included, 52.2% (95% CI, 52.0%-52.4%) were out of the labor market and 63.1% (95% CI, 62.9%-63.3%) were married. In 2010, 94% of all veterans and 94% of veterans younger than age 65 years reported having health insurance coverage on the American Community Survey. Insurance enrollment increased over time, and by 2020, 97% of all veterans and 95% of veterans younger than 65 years reported having health insurance coverage on the American Community Survey. Insurance enrollment estimates were similar across the surveys. Approximately one-third of veterans reported being enrolled in VA health coverage. Of those who enrolled in VA insurance, more than 75% had more than 1 form of coverage, with Medicare and private insurance being the most common second insurance sources. VA insurance enrollment was negatively associated with income and health status. Veterans without insurance tended to be unemployed and younger.

**CONCLUSIONS AND RELEVANCE:**

This study of veterans who responded to 4 national surveys found that veterans enrolled in VA health coverage had high rates of dual coverage. Further legislative efforts to increase access without recognizing the high rates of dual coverage may yield unintended consequences, such payer shifting.

## Introduction

Health insurance can streamline access to care, thereby improving well-being and health.^1–3^ In the US, major health insurance changes over the past decade include the Patient Protection and Affordable Care Act and Medicaid expansion.^4–8^ Yet, the federal government’s role in financing health insurance has stoked controversy in Congress, with much of that discussion focused on Medicare and Medicaid. What is notably missing from those debates is health care funding for the military and veterans. A 2023 Congressional Budget Office report noted that the President’s 2024 budget included $551 billion for military compensation, with more money going to Department of Veterans Affairs (VA) ($321 billion) than to the Department of Defense ($230 billion). The Congressional Budget Office further highlighted the increase in spending, stating “Since 2000, the total budget for military compensation has been rising steadily, even though the number of military personnel and veterans has been declining. Spending by VA has accounted for most of that increase.…”^9^

Sharp increases in VA spending should not surprise Congress, because they passed bills that expanded VA health care coverage, including the Choice Act of 2014 and the VA MISSION Act of 2018. Both pieces of legislation were designed to make it easier for veterans to access care and to hold VA accountable for mismanagement that delays care. These laws led to large increases in VA-purchased care, in which the VA pays for care that veterans obtain at non-VA institutions, with no changes in mortality, raising the question whether the increase in VA-purchased care truly addressed prior access problems and unmet needs.^10,11^

To understand and inform legislation on access to VA care, it is essential to examine how insurance coverage for US veterans has changed over time, given that insurance coverage enhances access by reducing the cost of care. Veterans can enroll in public and commercial health insurance and are also eligible to enroll in health insurance from the VA, providing an additional layer of health coverage. More than 9 million veterans are enrolled in VA health insurance, providing them options to receive VA-provided or VA-purchased care. Prior research has examined health insurance trends among veterans, focusing on the period prior to the Choice Act.^12–14^ In this study, we investigated veteran health insurance coverage rates and insurance sources from 2010 to 2021. We triangulated data across 3 nationally representative surveys, along with a survey of VA enrollees, to provide insight into how veterans’ health insurance coverage has evolved over time and how policies under debate might impact VA spending.

## Methods

This cross-sectional study was approved by the Palo Alto Research and Development Office and the Stanford University Institutional Review Board. Participants provided informed consent before responding to any of the included surveys. We followed the Strengthening the Reporting of Observational Studies in Epidemiology (STROBE) reporting guideline for cross-sectional studies.

### Data Sources

We analyzed data from the National Health Interview Survey (NHIS; 377 856 adults, 9% veterans), the American Community Survey (ACS; 27 000 161 adults, 8% veterans), and the Behavioral Risk Factor Surveillance System (BRFSS; 4 612 289 adults, 12% veterans). Each of these surveys is national in scope and conducted annually. We also analyzed data from the annual VA Survey of Enrollees (SOE; 535 394 adults), a survey designed to be representative of veterans enrolled in the VA health care system. We extracted ACS and SOE data starting in 2010 and NHIS and BRFSS data starting in 2011. The ACS data ended in 2020, while the other surveys ended in 2021.

### Variable Definition

The NHIS, ACS, and BRFSS asked respondents whether they had ever served in the US military, although the exact wording varied by survey and over time. We included all respondents who reporting having served in the US military. The surveys also asked respondents whether they were covered by health insurance, and all surveys except the BRFSS inquired about the type of coverage held (Medicaid, Medicare, Medicare Advantage, Private, TRICARE, and VA). We extracted the information on health insurance coverage, making it as consistent as possible, given response options.

From the ACS and NHIS surveys, we identified veterans who reported being covered or enrolled in VA health coverage. Again, there was variation in questionnaire wording, with the 2020 ACS asking whether the person was currently covered by any health insurance, with 1 option being “VA (enrolled for VA health care).” In contrast, the NHIS asked several questions to identify VA enrollment. We followed survey recommendations, when available, to best estimate VA enrollment. Dual-use was defined as being covered by non-VA health insurance while also enrolled in the VA.

To compute VA reliance, we used the SOE and computed the proportion of self-reported outpatient visits or trips that were made to VA health centers relative to all outpatient visits in the past calendar year. We extracted self-reported health status (excellent, very good, good, fair, or poor) from the NHIS to understand enrollment patterns by health.

Across all surveys, we extracted demographic information, including self-reported employment status, race (African American or Black, Asian, White, and other [eg, American Indian or Alaska Native, Native Hawaiian or Other Pacific Islander, and other race not specified]), ethnicity (Hispanic or not), and age. We use the term *Hispanic* to refer to someone who self-identified as Hispanic, Latinx, or Spanish origin.

We analyzed health insurance coverage for veterans over time. We stratified the analysis by age, given veterans aged 65 years and older had nearly universal coverage. We also compared coverage rates for veterans by self-reported health status and household income. Finally, we compared veterans by race and ethnicity, groups historically thought of as being at increased risk for having limited access to care.

### Statistical Analysis

We calculated the weighted mean coverage rates by using survey weights. ACS and NHIS survey weights produce nationally representative estimates, while the BRFSS weights ensure representation at the state level. The SOE weights were designed to make the sample representative of VA enrollees. We did not conduct regression analysis given our goal was to generate population estimates without conditioning on specific covariates. No attempt was made to provide causal estimates. All analyses were conducted using Stata software version 18 (StataCorp). Data were analyzed from October 2023 to June 2024.

## Results

### Sample Characteristics

A total of 3 644 614 survey respondents (mean [SE] age, 60 [0.04] years; 91.3% [95% CI, 91.2%-91.5%] male) were included. eTable 1 in Supplement 1 shows the sample characteristics for the combined surveys. Hispanic or Latino ethnicity was reported by 6.4% (95% CI, 6.3%-6.5%); 12.1% (95% CI, 12.0%-12.3%) of respondents were African American or Black, 1.7% (95% CI, 1.7%-1.8%) of respondents were Asian, and 82.3% (95% CI, 82.2%-82.5%) of respondents were White. Most veterans (52.2% [95% CI, 52.0%-52.4%]) reporting being retired or out of the labor market, with 44.4% (95% CI, 44.2%-44.6%) reporting being employed and 3.4% (95% CI, 3.4%-3.5%) being unemployed. Most veterans (63.1% [95% CI, 62.9%-63.3%]) reported being married. eTable 1 in Supplement 1 shows sample characteristics by survey; respondents in the SOE had older respondents, fewer self-reported White respondents, and more respondents who reported being employed than respondents in the other surveys.

### Health Insurance Coverage Among Veterans

**Figure 1.**
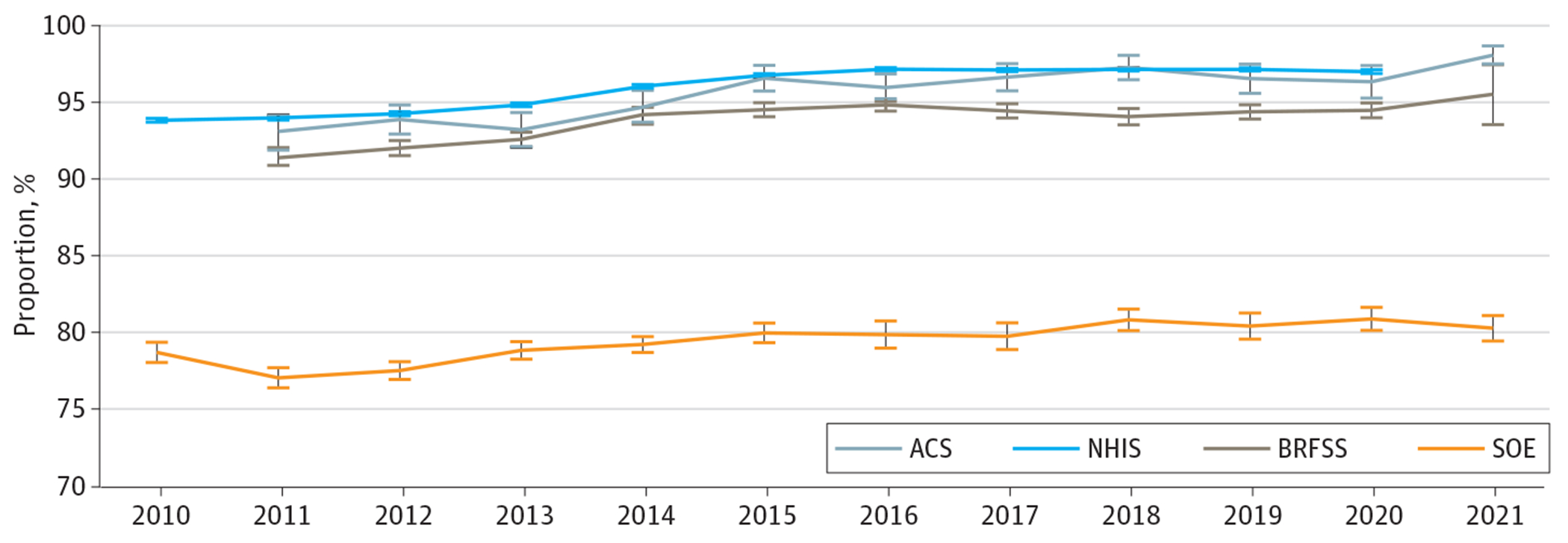
Share of Veterans Reporting Any Health Insurance Coverage by Survey Source The Department of Veterans Affairs (VA) Survey of Enrollees (SOE) represents any insurance, excluding VA coverage. Each analysis is weighted with respective survey weights. Whiskers represent 95% CIs. ACS indicates American Community Survey; BRFSS, Behavioral Risk Factor Surveillance Survey; NHIS, National Health Interview Survey.

Since 2010, 94% of veterans reported having health insurance from any source. As shown in Figure 1, there was some variation across surveys, with rates of insurance coverage increasing from 2010 to 2015 and relatively stable since 2015. More than half of veterans (52%) reported having private insurance, and approximately one-half of the veterans (49%) reported Medicare coverage (Table). Among veterans younger than age 65 years, the overall results show similar trends, with 94% veterans reporting being enrolled in insurance in 2021. eFigure 1 in Supplement 1 shows trends over time for veterans younger than age 65 years and shows the increased rates of insurance between 2014 and 2016, consistent with the implementation of the Patient and Protection and Affordable Care Act.

Overall, private insurance was the most common source, according to the NHIS and ACS surveys, at 52.8% (95% CI, 51.5%-54.1%) of veterans in NHIS and 59.4% (95% CI, 59.2%-59.6%) of veterans in ACS (Table). In these 2 surveys, Medicare was the next most common source of insurance, followed by VA. The SOE, which samples veterans who used the VA, had rates that differed from NHIS and ACS (Table). The overall results were similar for veterans younger than 65 years, except for substantially lower Medicare enrollment, as expected.

Dual coverage was common among VA enrollees (Table). According to the SOE, among VA enrollees, approximately 79.5% (95% CI, 79.0%-80.0%) had another source of health insurance, exclusive of VA coverage. The ACS had dual coverage at 88.6% (95% CI, 88.3%-88.9%), while NHIs’s estimate was 77.9% (95% CI, 75.0%-78.9%). Dual coverage was lower for veterans younger than 65 years, with some variation among the surveys (Table).

The composition of health insurance coverage among veterans changed over time. Over the decade of survey collection, there was a slow decline in private insurance, accompanied by a slow rise in Medicare enrollment, including Medicare Advantage (eFigures 2–5 in Supplement 1). Medicaid coverage was stable over time and relatively uncommon (<10% of respondents). The compositional change in insurance partly reflected the large cohort of Vietnam-era veterans becoming age-eligible for Medicare.

### Enrollment in VA

While almost all veterans had health insurance, most veterans did not enroll in VA health coverage according to NHIS and ACS data (Table). More than half of veterans reported having private insurance in the NHIS and ACS surveys, and those without private insurance were much more likely to enroll in VA. Medicare was the next most common source of insurance, reported by approximately half of veterans.

**Figure 2.**
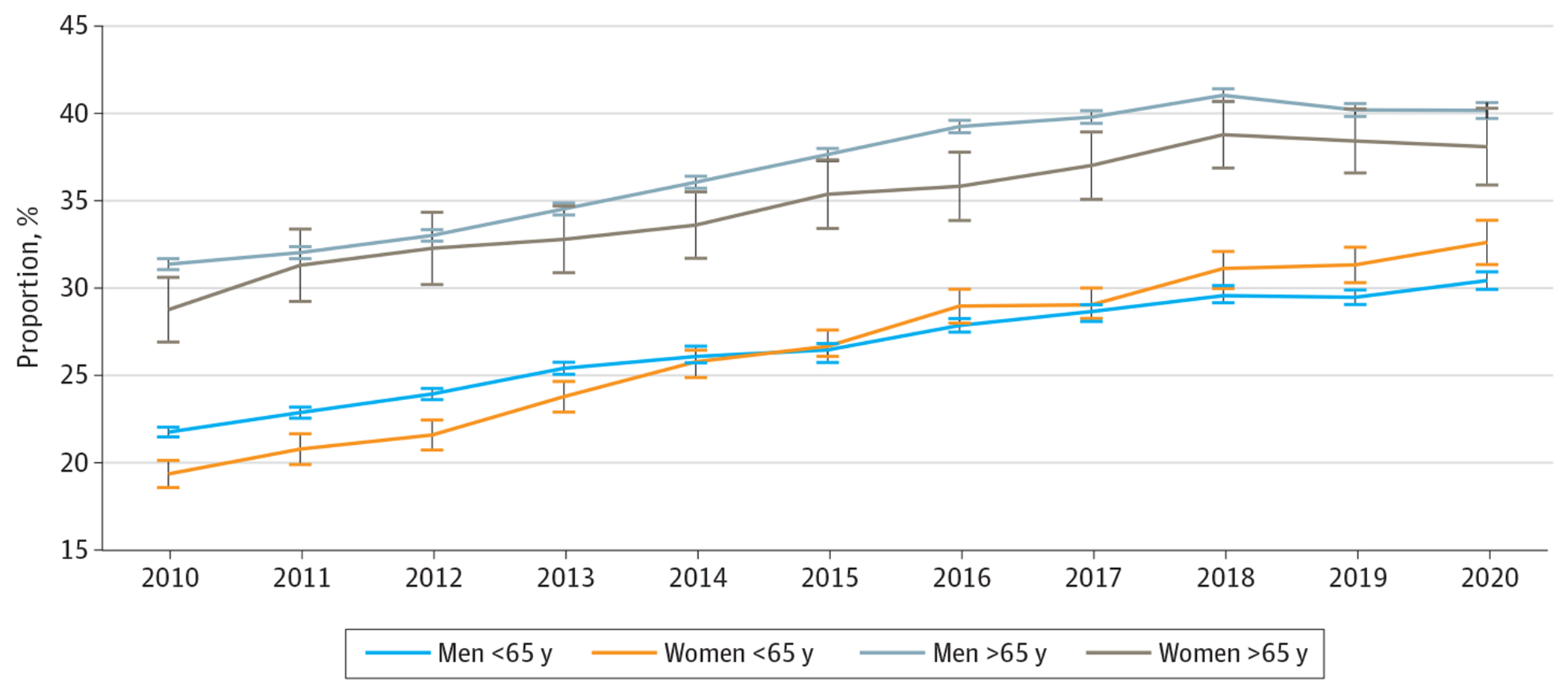
Share of Veterans Reporting Department of Veterans Affairs VA Enrollment Over Time in the American Community Survey Analyses were weighted to be nationally representative. Whiskers represent 95% CIs.

Enrollment in the VA increased over time for both men and women. In 2010, 20.6% (95% CI, 19.9%-21.3%) of women veterans were enrolled in the VA, compared with 25.7% (95% CI, 25.5%-25.9%) of men. By 2020, the gap between men and women shrank, with 33.6% (95% CI, 32.5%-34.7%) of women and 35.3% (95% CI, 34.9%-35.6%) of men enrolled. Figure 2 shows the trends for men and women stratified by age over time. VA enrollment increased faster for women younger than 65 years, such that by 2020, a higher percentage of women were enrolled in VA (32.6% [95% CI, 29.9%-30.8%]) compared with men (30.4% [95% CI, 31.3%-33.8%]).

### Reliance on VA

**Figure 3.**
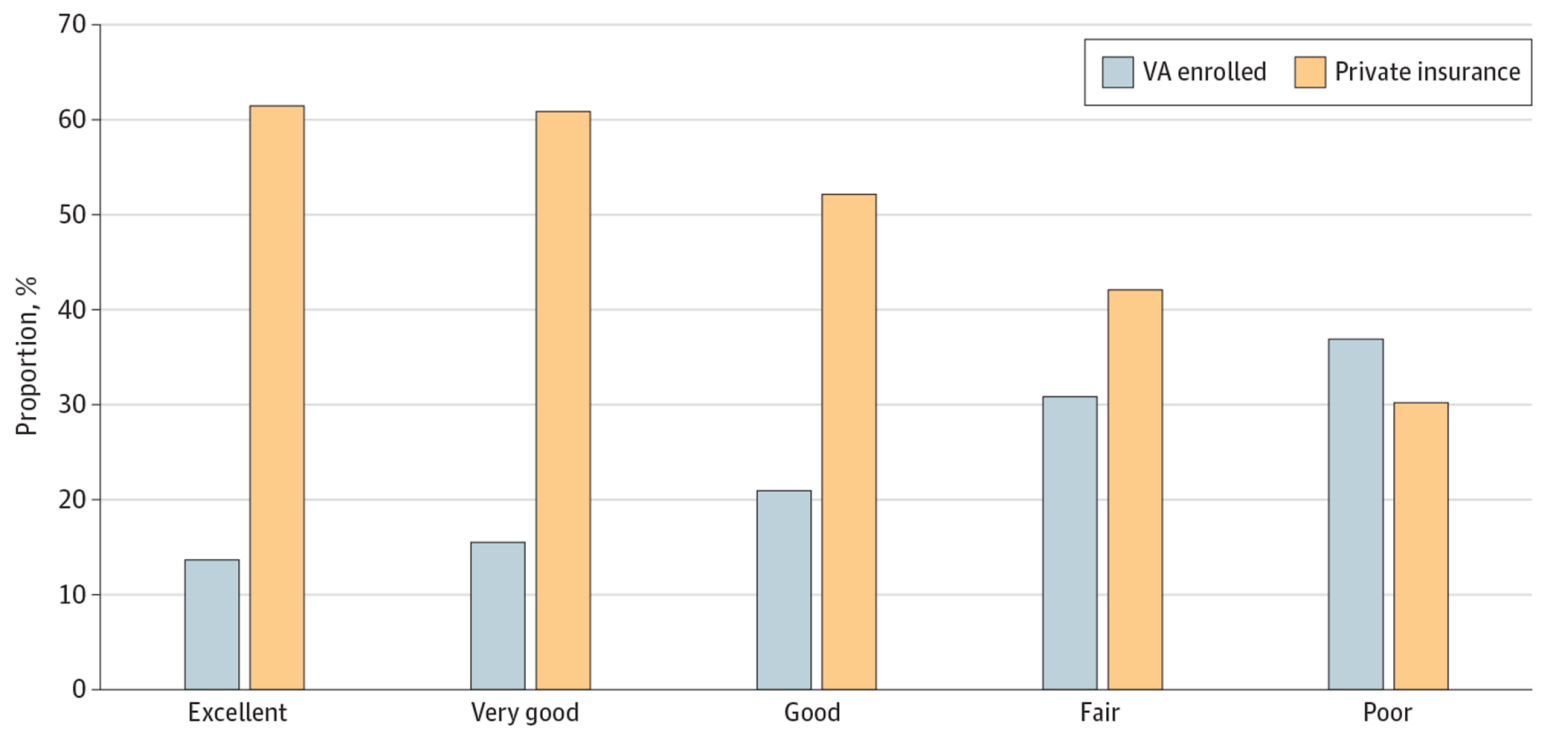
Trends in Coverage for Veterans by Self-Reported Health Status in the National Health Interview Survey Data on veteran employment status were weighted to be nationally representative. VA indicates Department of Veterans Affairs.

Individuals with higher VA reliance reported lower health status and lower income. Veterans who self-reported having excellent health had considerably lower VA enrollment rates than veterans who self-report their health as good, fair, or poor (Figure 3). Overall, veterans in poor health were approximately 3 times more likely to enroll in the VA than those in excellent health. In contrast, decrements in health status were negatively associated with private insurance coverage. Veterans with less income were more likely to rely on the VA for more of their health care utilization (eFigure 6 in Supplement 1).

### Coverage by Employment Status, Race and Ethnicity, and Age

Unemployed veterans consistently reported the lowest levels of insurance coverage, fluctuating between 60% and 90% by year, depending on the survey. Retired veterans and those out of the labor force had the highest coverage rates (>90%), followed closely by employed veterans (eFigures 7–9 in Supplement 1).

Veterans in different race and ethnic groups all reported high levels of insurance coverage (91%-99%). The changes in proportion of veterans reporting any insurance for all racial groups are shown in eTable 2 and eFigures 11 to 13 in Supplement 1. The ACS, with its larger samples, had a steady pattern: Asian and White veterans consistently had the highest overall coverage rates, followed by Black veterans, and then the other category. There were no large discrepancies by racial or ethnic groups.

Insurance coverage increased with age, and insurance coverage improved between 2010 and 2021 across all age groups. The biggest change over time was seen among younger veterans. In 2011, 79.3% (95% CI, 73.0%-85.6%) of veteran respondents aged 25 to 34 years reported having any insurance in the NHIS. By 2017, this was 92.6% (95% CI, 88.4%-96.8%) and remained over 90% through 2021. Across all 3 surveys, coverage for veterans aged 65 years and older was near universal (99%-100%) (eTable 3 in Supplement 1).

## Discussion

The findings of this cross-sectional study provide important insights into the potential policy effects of further legislative efforts to expand access to VA health care. First, efforts to expand VA coverage to address unmet needs must consider how changes will affect veterans with dual coverage. More than 75% of veterans enrolled in VA health coverage were covered by multiple sources. Older veterans had dual coverage through Medicare, while younger veterans more frequently reported dual coverage through private insurance.

More than 9 million veterans enroll in VA health coverage, and the VA provides care to more than 6 million enrollees nationally. The VA is the largest integrated health care system in the US, with 172 medical centers and more than 1100 outpatient clinics. In the past decade, Congress passed legislation to expand health care access for veterans, and Congress continues to debate measures that would further expand access.^15^ The VA has authority to seek reimbursement from private insurers when providing a veteran non–service connected care. However, no such arrangement exists between the VA and Medicare. Historically, veterans who were enrolled in VA health coverage and also in Medicare were able to choose whether to go to a VA hospital or a commercial hospital using their Medicare coverage. That changed with the Choice Act of 2014 and the VA MISSION Act of 2018.^16^ Both acts expanded eligibility for VA purchased care, such that an eligible veteran could go to a commercial hospital and the VA would be responsible for paying. Thus, veterans who are newly eligible for VA-purchased care can choose whether to use their VA benefits or their Medicare benefits. A 2023 study^17^ using all payer claims data in NY state reported that veterans with Medicare coverage switched their payer from Medicare to VA after the VA MISSION Act.^18^

Payer shifting is rational from a veteran’s perspective because it reduces their out-of-pocket costs, but it may not be optimal from a policy perspective. One problem is that the VA, unlike Medicare, faces a fixed annual budget set by Congress. VA medical centers are not allowed to save money from year to year, so when budgets are increasingly uncertain and volatile, a common response is for VA facility leaders to enact hiring freezes, which can jeopardize valuable programs. Such responses have been seen previously.^18,19^ Another problem is that the VA has not been given the same tools to address financial risk that have been given to health plans overseen by the Center for Medicare & Medicaid Services (CMS). The Choice and VA MISSION Acts expanded the VA’s use of VA-purchased care, but the VA must adhere to VA contracting requirements. The VA cannot negotiate on quality or price because the acts link VA prices to mean Medicare fee-for-service prices,^16^ thereby incentivizing volume rather than value. Thus, these VA contracts, as currently operating, are a liability for the VA.

The VA Center for Care and Payment Innovation (CCPI), recently authorized by Congress, allows the VA to experiment with payment innovations in health care, much like the CMS Innovation Center. To achieve its goal of “Strengthening care for veterans now and in the future,”^20^ CCPI will need to focus on veterans with dual VA and Medicare coverage, given that 49% of veterans are older than 65 years and another 18% of veterans will be eligible for Medicare in the next decade.^21^ Top of the agenda should be efficient payment policies between the VA and CMS. Currently, tax payers are making substantial duplicate payments to VA to Medicare Advantage each year,^22^ and payment policies could help VA and tax payers.

CCPI will also then need to develop meaningful access metrics. Currently, the VA is fixated on wait times, including a real-time dashboard, as a measure of access.^23^ That might appear useful for a veteran who is trying to decide whether to use VA-purchased or VA-provided care, until the veteran realizes that no commercial health care organization provides comparable wait time data. Moreover, for many services, wait times reflect convenience, rather than medical appropriateness. Finally, CCPI will need to test alternative payment models that reward improved outcomes, while not hindering equitable access. Fortunately, CCPI can learn from CMS Innovation Center’s past work.^24^

### The VA as a Safety Net

The VA is part of the US safety net,^25^ and in many ways, the VA safety net appears to be working as intended. Consistently over time, veterans with worse health status and lower household income were more likely to enroll in VA for health care. This could reflect patient preferences and needs, but it could also reflect the priority status that VA gives to veterans with a service-connected disability and low household income, exempting them from copayments.^26^

A closer inspection shows a safety net with some holes, although those holes may be shrinking. Unemployed veterans are at greater risk of not having health insurance. However, some of these veterans may not be eligible for VA benefits. Historically, VA eligibility was open to honorably discharged veterans. Yet, an increasing number of studies show that veterans at higher risk for poor outcomes, such as suicide, are not eligible for VA care because they were discharged other than honorably, possibly because of health conditions acquired while in the military.^27^ The Compact Act of 2020 partly addressed this gap by making VA responsible for any veteran who arrives under acute psychiatric distress at any hospital.^28^ In 2024, the VA further extended eligibility for veterans who were previously ineligible for enrollment.^29^ Future research is needed to understand the outcomes of these new rules.

### Limitations

This study has some limitations. We examined national survey data from 2010 to 2021. Variation within surveys over time and across surveys limited our ability to pull detailed information that would help us elucidate some of the broader changes in insurance coverage. We have highlighted specific surveys when appropriate, including tabulations from other surveys in technical appendices. Analyses of survey data also introduce potential biases due to nonresponse or uncertainty from small samples. One of the benefits of pooling data across multiple surveys is the added confidence from seeing very similar results. Furthermore, we also did not conduct any statistical tests. Our goal was to track population trends, without conditioning on covariates, and then show how the trends are intertwined with policies. No attempt was made to draw causal connections, and future policies that rely on causal assumptions should look to research that explicitly uses methods appropriate for causal identification.

## Conclusions

The findings of this cross-sectional study underscore the importance of the VA as a stable source of coverage for some veterans, although most veterans do not enroll in VA. Understanding the composition of insurance coverage for veterans is critical for policymakers who are seeking to help veterans access care while simultaneously conserving federal costs and improving system efficiencies. Further efforts to increase access, without recognizing the potential for payer shifting, may just increase VA costs without any corresponding improvements in health.

## Supplementary Material

Supplement 1 **eTable 1.** Sample Characteristics
**eFigure 1.** Trends in Coverage for Veterans Under Age 65
**eFigure 2.** Trends in Private Coverage for Veterans
**eFigure 3.** Trends in Any Medicare and Medicare Advantage (MA) Coverage for Veterans.
**eFigure 4.** Trends in TRICARE Coverage for Veterans
**eFigure 5.** Trends in Medicaid Coverage for Veterans
**eFigure 6.** Share of Veterans Reliant on VA by Household Income (SOE)
**eFigure 7.** Share of Veterans Reporting Any Insurance Coverage by Employment Status (ACS)
**eFigure 8.** Share of Veterans Reporting Any Insurance Coverage by Employment Status (BRFSS)
**eFigure 9.** Share of Veterans Reporting Any Insurance Coverage by Employment Status (NHIS)
**eFigure 10.** Insurance Coverage Rates for Veterans by Race (ACS)
**eTable 2.** Veteran Insurance Coverage by Race and Ethnicity (ACS)
**eFigure 11.** Insurance Coverage Rates for Veterans by Race (ACS)
**eFigure 12.** Insurance Coverage Rates for Veterans by Race (BRFSS)
**eFigure 13.** Insurance Coverage Rates for Veterans by Race (NHIS)
**eTable 3.** Veteran Insurance Coverage by Age

Supplement 2 Data Sharing Statement

## Figures and Tables

**Table.** Insurance Coverage Rates Among Veterans by VA Enrollment Status, 2019-2021

Enrollment	Veterans, % (95% CI)					

	Overall			Age <65 y		
	NHIS (n = 8221)	ACS (n = 382 108)	SOE (n = 129 028)^a^	NHIS (n = 3519)	ACS (n = 177 832)	SOE (n = 35 055)^a^
**Total Sample**						

VA	32.0 (30.8-33.2)	34.8 (34.6-35)	85.8 (85.5-86.2)	31.5 (29.7-33.3)	30.2 (29.9-30.5)	84.5 (83.8-85.1)

Private	52.8 (51.5-54.1)	59.4 (59.2-59.6)	26.4 (25.9-26.8)	58.1 (56.2-60)	61.5 (61.2-61.8)	35.3 (34.4-36.1)

Medicare	49.1 (47.9-50.4)	48.9 (48.7-49.1)	51.4 (50.9-51.9)	5.4 (4.5-6.2)	5.4 (5.3-5.5)	12.9 (12.4-13.4)

Tricare	22.1 (21-23.1)	23.8 (23.6-24)	22.5 (22.1-22.9)	25.0 (23.3-26.6)	29.5 (29.2-29.7)	26.7 (25.9-27.4)

Medicaid	4.0 (3.5-4.5)	9.3 (9.2-9.4)	7.2 (7-7.4)	5.6 (4.7-6.5)	8.8 (8.6-9)	5.9 (5.5-6.3)

Dual with VA^b^	23.0 (21.9-24)	30.9 (30.7-31.1)	67.8 (67.3-68.2)	18.4 (16.9-19.8)	15.9 (15.6-16.1)	11.0 (10.6-11.5)

**Not enrolled in VA**						

Private	58.8 (57.2-60.4)	61.9 (61.6-62.1)	36.7 (35.3-38.1)	63.7 (61.3-66.1)	65.3 (64.9-65.7)	47.0 (44.8-49.2)

Medicare	48.5 (46.9-50.1)	44.3 (44-44.5)	47.4 (46.1-48.7)	4.2 (3.2-5.1)	3.7 (3.5-3.8)	11.3 (10.1-12.6)

Tricare	18.3 (17-19.5)	21.9 (21.6-22.1)	26.2 (25-27.4)	21.2 (19.2-23.2)	29.3 (29-29.7)	30.3 (28.3-32.3)

Medicaid	4.4 (3.7-5.1)	8.0 (7.8-8.1)	8.3 (7.6-8.9)	6.0 (4.8-7.2)	7.6 (7.4-7.8)	6.3 (5.4-7.3)

**Enrolled in VA**						

Private	38.9 (36.8-41.1)	54.7 (54.4-55.1)	24.7 (24.2-25.2)	43.1 (39.8-46.4)	52.6 (52-53.2)	33.1 (32.2-34)

Medicare	48.1 (45.9-50.3)	57.6 (57.2-58)	52.0 (51.5-52.5)	7.1 (5.6-8.7)	9.4 (9.1-9.7)	13.2 (12.6-13.7)

Tricare	34.4 (32.3-36.6)	27.4 (27.1-27.7)	22.0 (21.5-22.4)	37.0 (33.7-40.2)	29.7 (29.2-30.2)	26.0 (25.2-26.8)

Medicaid	3.1 (2.4-3.8)	11.7 (11.5-11.9)	7.0 (6.7-7.2)	4.4 (3.2-5.6)	11.5 (11.2-11.9)	5.8 (5.4-6.2)

Dual with VA^b^	76.9 (75-78.9)	88.6 (88.3-88.9)	79.5 (79-80)	61.9 (58.7-65.1)	76.3 (75.8-76.8)	65.5 (64.6-66.4)

Abbreviations: ACS, American Community Survey; NHIS, National Health Interview Survey; SOE, VA Survey of Enrollees; VA, Department of Veterans Affairs.

a The SOE sampling frame targets veterans who were enrolled in VA.

b Represents VA enrollment plus another source of insurance.

## Data Availability

See Supplement 2.

## Key Points

### Question

How has insurance coverage changed among veterans from 2010 to 2021?

### Findings

This cross-sectional study using national survey data from 3 644 614 veterans from 2010 to 2021 found that more than 90% of veterans reported having health insurance coverage, and of those enrolled in VA health coverage, more than 75% had another source of coverage. Dual coverage remained high even among veterans younger than age 65 years.

### Meaning

These findings suggest that veterans were generally well insured and future legislation to improve access will need to target specific populations, otherwise legislative efforts may result in unintended consequences, such as payer shifting.
