# Supplementary material for: Health Insurance Enrollment Among US Veterans, 2010-2021
Source: JAMA Netw Open. Author manuscript; Available in PMC 2024 Oct 28. (PMC11514437; doi:10.1001/jamanetworkopen.2024.30205)
Supplement: Supplement 1 — eTable 1. Sample Characteristics eFigure 1. Trends in Coverage for Veterans Under Age 65 eFigure 2. Trends in Private Coverage for Veterans eFigure 3. Trends in Any Medicare and Medicare Advantage (MA) Coverage for Veterans. eFigure 4. Trends in TRICARE Coverage for Veterans eFigure 5. Trends in Medicaid Coverage for Veterans eFigure 6. Share of Veterans Reliant on VA by Household Income (SOE) eFigure 7. Share of Veterans Reporting Any Insurance Coverage by Employment Status (ACS) eFigure 8. Share of Veterans Reporting Any Insurance Coverage by Employment Status (BRFSS) eFigure 9. Share of Veterans Reporting Any Insurance Coverage by Employment Status (NHIS) eFigure 10. Insurance Coverage Rates for Veterans by Race (ACS) eTable 2. Veteran Insurance Coverage by Race and Ethnicity (ACS) eFigure 11. Insurance Coverage Rates for Veterans by Race (ACS) eFigure 12. Insurance Coverage Rates for Veterans by Race (BRFSS) eFigure 13. Insurance Coverage Rates for Veterans by Race (NHIS) eTable 3. Veteran Insurance Coverage by Age [file NIHMS2026864-supplement-Supplement_1.pdf]

Supplemental Online Content

Wagner TH, Schmidt A, Belli F, et al. Health insurance enrollment among US veterans, 2010-2021. *JAMA Netw Open*. 2024;7(8):e2430205.
doi:10.1001/jamanetworkopen.2024.30205

eTable 1. Sample Characteristics
eFigure 1. Trends in Coverage for Veterans Under Age 65
eFigure 2. Trends in Private Coverage for Veterans
eFigure 3. Trends in Any Medicare and Medicare Advantage (MA) Coverage for Veterans.
eFigure 4. Trends in TRICARE Coverage for Veterans
eFigure 5. Trends in Medicaid Coverage for Veterans
eFigure 6. Share of Veterans Reliant on VA by Household Income (SOE)
eFigure 7. Share of Veterans Reporting Any Insurance Coverage by Employment Status (ACS)
eFigure 8. Share of Veterans Reporting Any Insurance Coverage by Employment Status (BRFSS)
eFigure 9. Share of Veterans Reporting Any Insurance Coverage by Employment Status (NHIS)
eFigure 10. Share of Veterans Reporting Any Insurance Coverage by Employment Status (NHIS)
eTable 2. Veteran Insurance Coverage by Race and Ethnicity (ACS)
eFigure 11. Insurance Coverage Rates for Veterans by Race (ACS)
eFigure 12. Insurance Coverage Rates for Veterans by Race (BRFSS)
eFigure 13. Insurance Coverage Rates for Veterans by Race (NHIS)
eTable 3. Veteran Insurance Coverage by Age

This supplemental material has been provided by the authors to give readers additional information about their work.

© 2024 Wagner TH et al. *JAMA Network Open.*

Table e1: Sample Characteristics

	NHIS<br>% (SE)	ACS<br>% (SE)	BRFSS<br>% (SE)	SOE<br>% (SE)	Overall<br>% (SE)
Age: Avg	60.8	59.7	59.1	61.3	60.0
	(0.1182)	(0.0155)	(0.0528)	(0.0482)	(0.0384)
Sex (% female)	9%	9%	9%	8%	9%
	(0.0019)	(0.0002)	(0.0008)	(0.0007)	(0.0006)
Hispanic	6%	6%	7%	7%	6%
	(0.0017)	(0.0002)	(0.0009)	(0.0007)	(0.0006)
White	84%	82%	82%	79%	82%
	(0.0025)	(0.0003)	(0.0012)	(0.001)	(0.0008)
Black/African-American	12%	12%	12%	12%	12%
	(0.0022)	(0.0003)	(0.001)	(0.0008)	(0.0007)
Asian	2%	2%	2%	1%	2%
	(0.0009)	(0.0001)	(0.0006)	(0.0004)	(0.0003)
Other	2%	4%	4%	7%	4%
	(0.0009)	(0.0002)	(0.0006)	(0.0006)	(0.0003)
Employment status					
Employed	46%	48%	43%	36%	44%
	(0.0035)	(0.0004)	(0.0013)	(0.0012)	(0.0011)
Unemployed	2%	3%	4%	5%	3%
	(0.0011)	(0.0001)	(0.0006)	(0.0006)	(0.0004)
Retired / out of labor force	52%	50%	52%	59%	52%
	(0.0035)	(0.0004)	(0.0013)	(0.0012)	(0.0011)
Marital status					
Married	65%	63%	62%	62%	63%
	(0.0031)	(0.0004)	(0.0013)	(0.0012)	(0.001)
Separated	2%	2%	2%	2%	2%
	(0.0008)	(0.0001)	(0.0004)	(0.0004)	(0.0003)
Divorced	12%	15%	14%	15%	14%
	(0.0019)	(0.0003)	(0.0009)	(0.0008)	(0.0006)
Widowed	7%	7%	9%	7%	8%
	(0.0014)	(0.0002)	(0.0006)	(0.0005)	(0.0005)
Married/Unmarried couple	13%	12%	14%	13%	13%
	(0.0023)	(0.0003)	(0.0011)	(0.001)	(0.0008)
unweighed sample size	34,068	2,474,156	615,142	521,248	3,644,614

Linearized standard errors in parentheses

© 2024 Wagner TH et al. *JAMA Network Open.*

eFigure 1. Trends in Coverage for Veterans under age 65

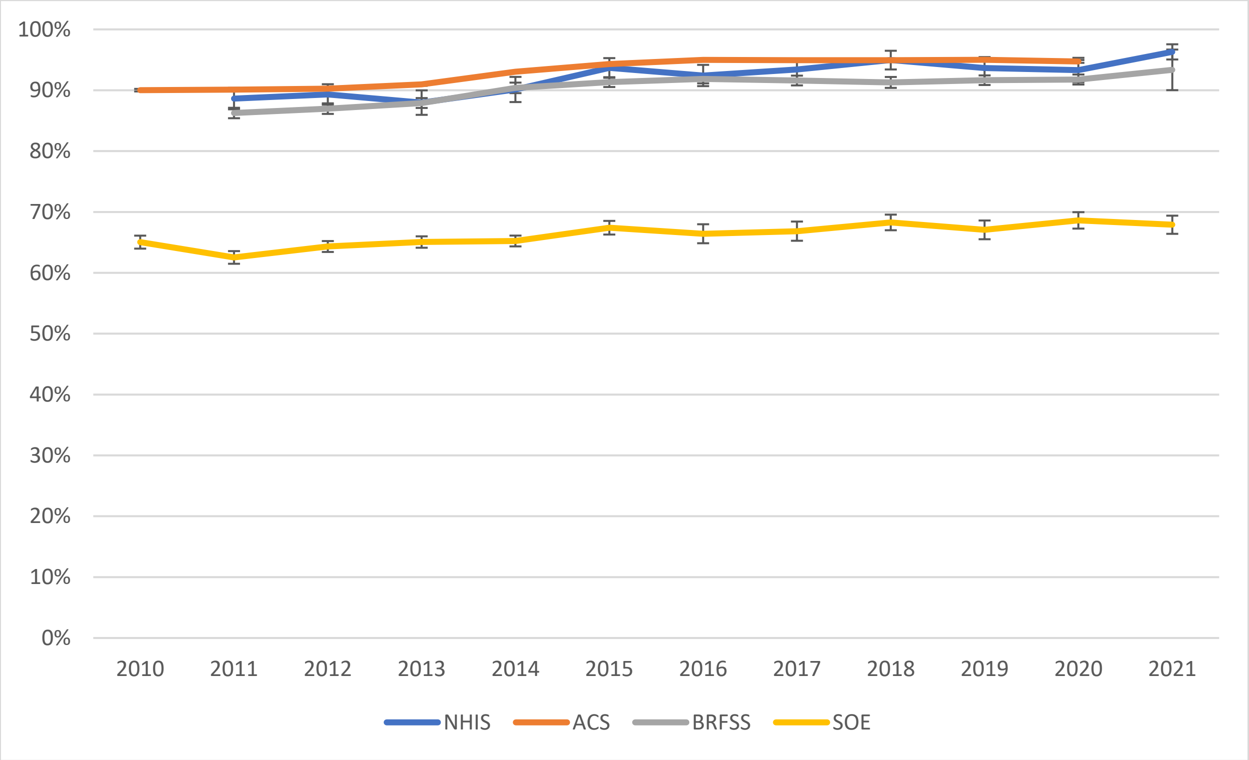

Notes: ACS is American Community Survey. NHIS is National Health Interview Survey. BRFSS is Behavioral Risk Factor Surveillance Survey. SOE is VA Survey of Enrollees. SOE represents any insurance, excluding VA coverage. Each analysis is weighted with respective survey weights. Whiskers represent 95% confidence intervals.

© 2024 Wagner TH et al. *JAMA Network Open.*

eFigure 2. Trends in Private Coverage for Veterans

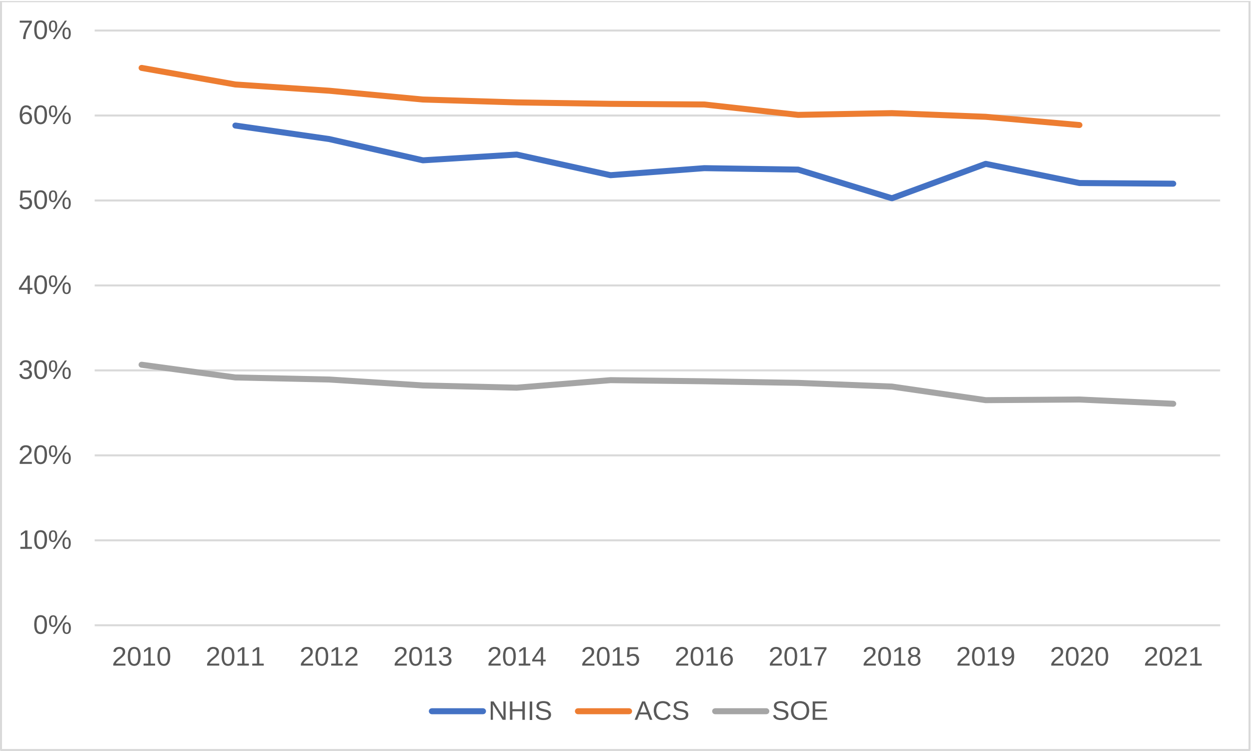

Note: NHIS is National Health Interview Survey. ACS is American Community Survey. SOE is VA Survey of Enrollees. The BRFSS survey was excluded because it does not ask about types of coverage held by respondents.

© 2024 Wagner TH et al. *JAMA Network Open.*

eFigure 3: Trends in Medicare Coverage for Veterans.

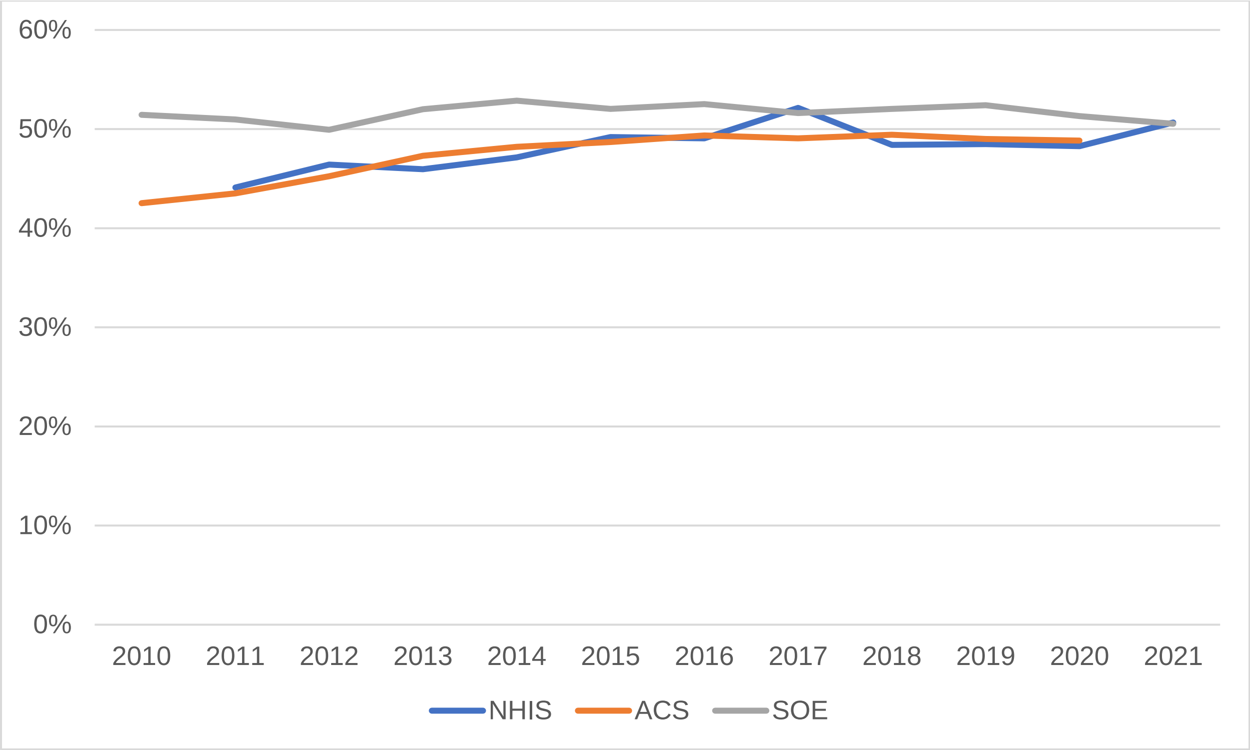

Notes: NHIS is National Health Interview Survey. ACS is American Community Survey. NHIS is National Health Interview Survey. SOE is VA Survey of Enrollees. The BRFSS survey was excluded because it does not ask about the type of coverage held by respondents.

© 2024 Wagner TH et al. *JAMA Network Open.*

eFigure 4: Trends in Medicare Advantage Coverage for Veterans

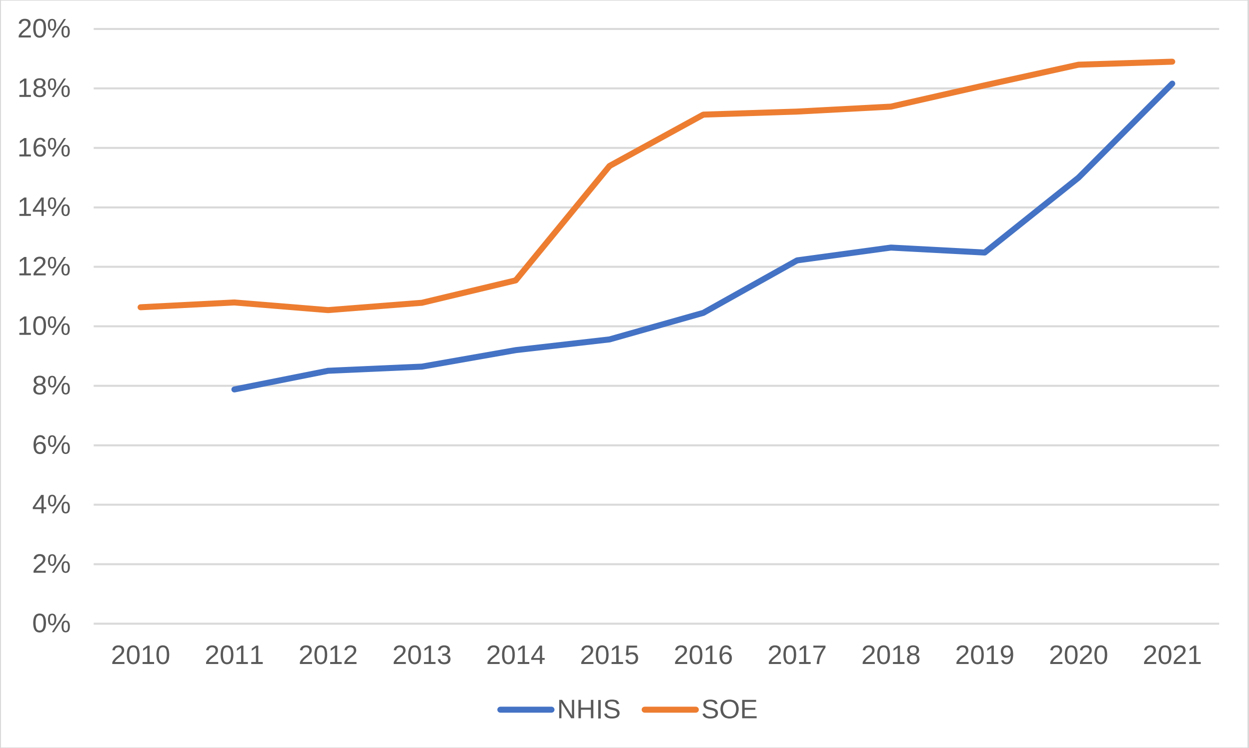

Notes: NHIS is National Health Interview Survey. SOE is VA Survey of Enrollees. The ACS and BRFSS survey were excluded because they do not ask about Medicare Advantage.

© 2024 Wagner TH et al. *JAMA Network Open.*

eFigure 5: Trends in Tricare Coverage for Veterans

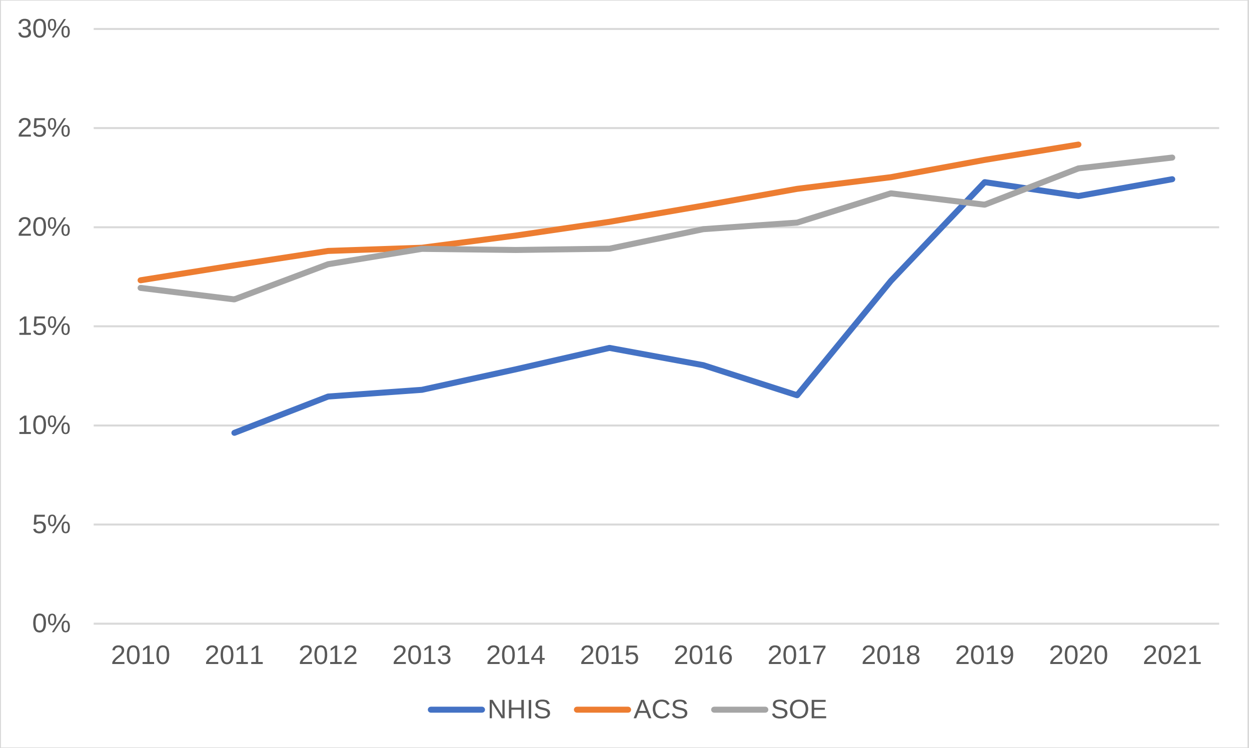

Notes: NHIS is National Health Interview Survey. ACS is American Community Survey. SOE is VA Survey of Enrollees. The BRFSS survey was excluded because it does not ask about the type of coverage held by respondents.

© 2024 Wagner TH et al. *JAMA Network Open.*

eFigure 6: Trends in Medicaid Coverage for Veterans

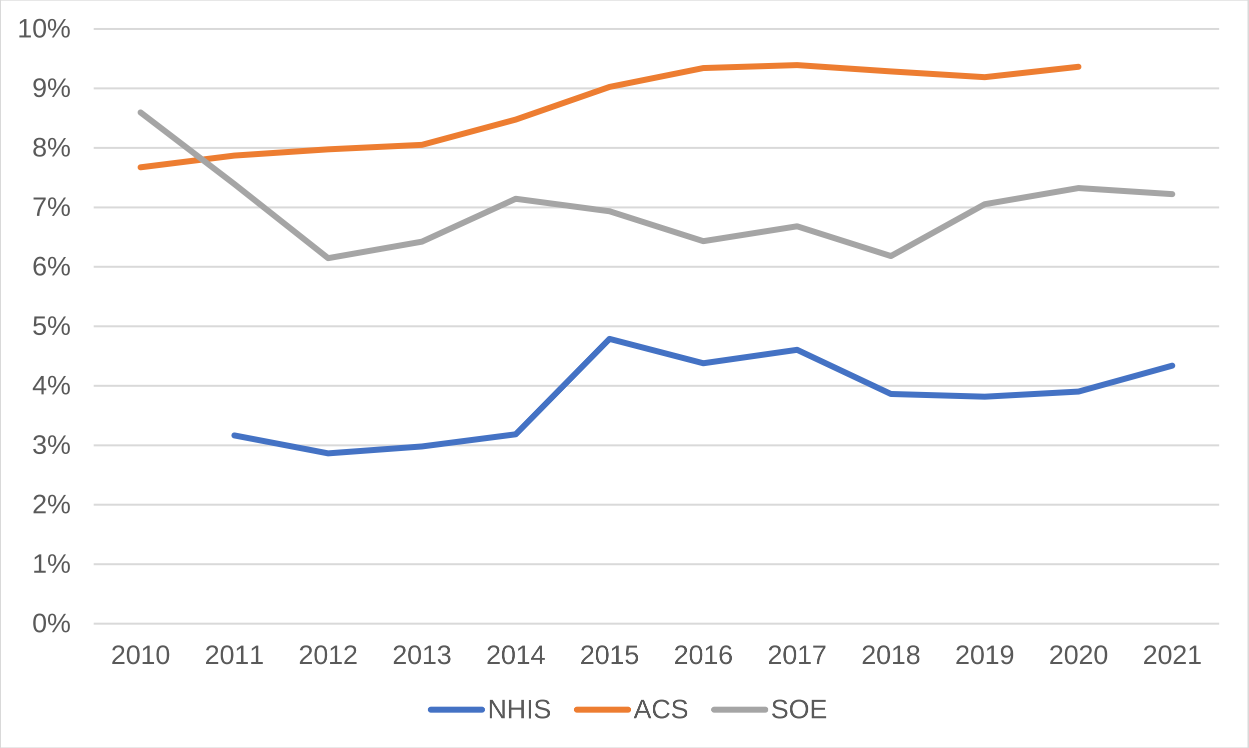

Notes: NHIS is National Health Interview Survey. ACS is American Community Survey. SOE is VA Survey of Enrollees. The BRFSS survey was excluded because it does not ask about the type of coverage held by respondents.

© 2024 Wagner TH et al. *JAMA Network Open.*

eFigure 7: Trends in Coverage for Veterans by Self-Reported Health Status (NHIS)

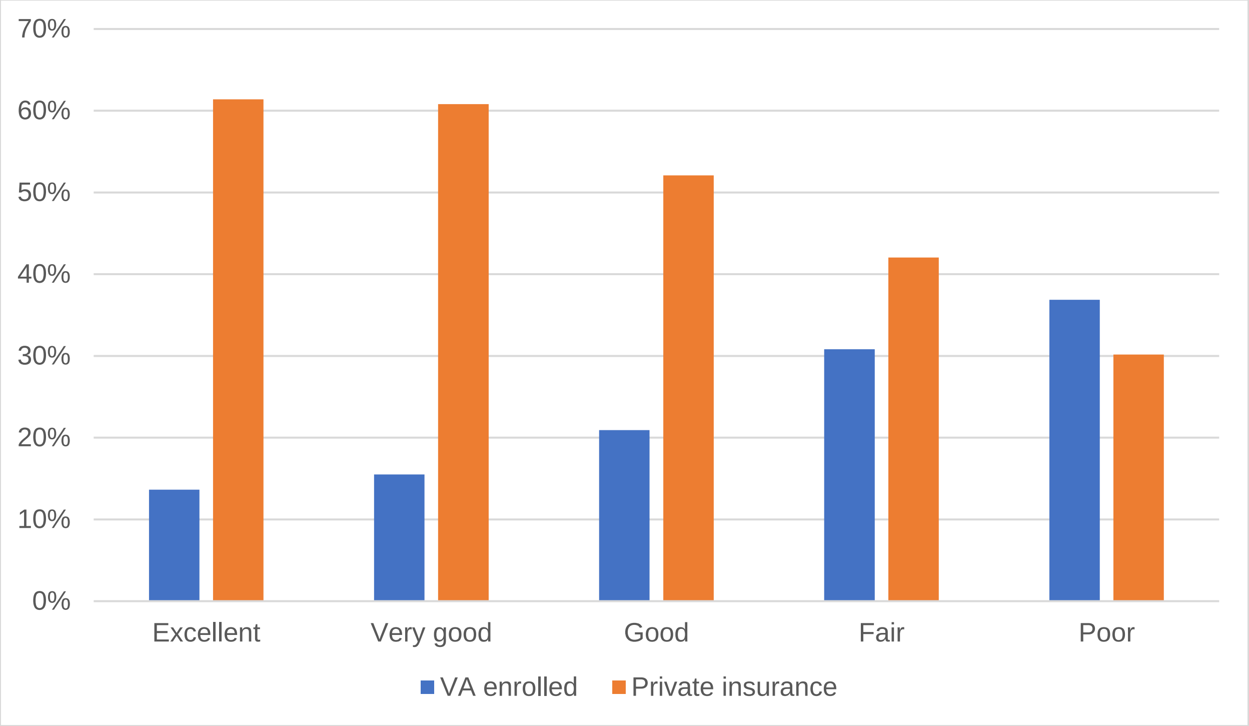

NHIS is National Health Interview Survey. NHIS data on Veteran employment status was weighted to be nationally representative.

© 2024 Wagner TH et al. *JAMA Network Open.*

eFigure 8: Share of Veterans Reporting Any Insurance Coverage by Employment Status (ACS)

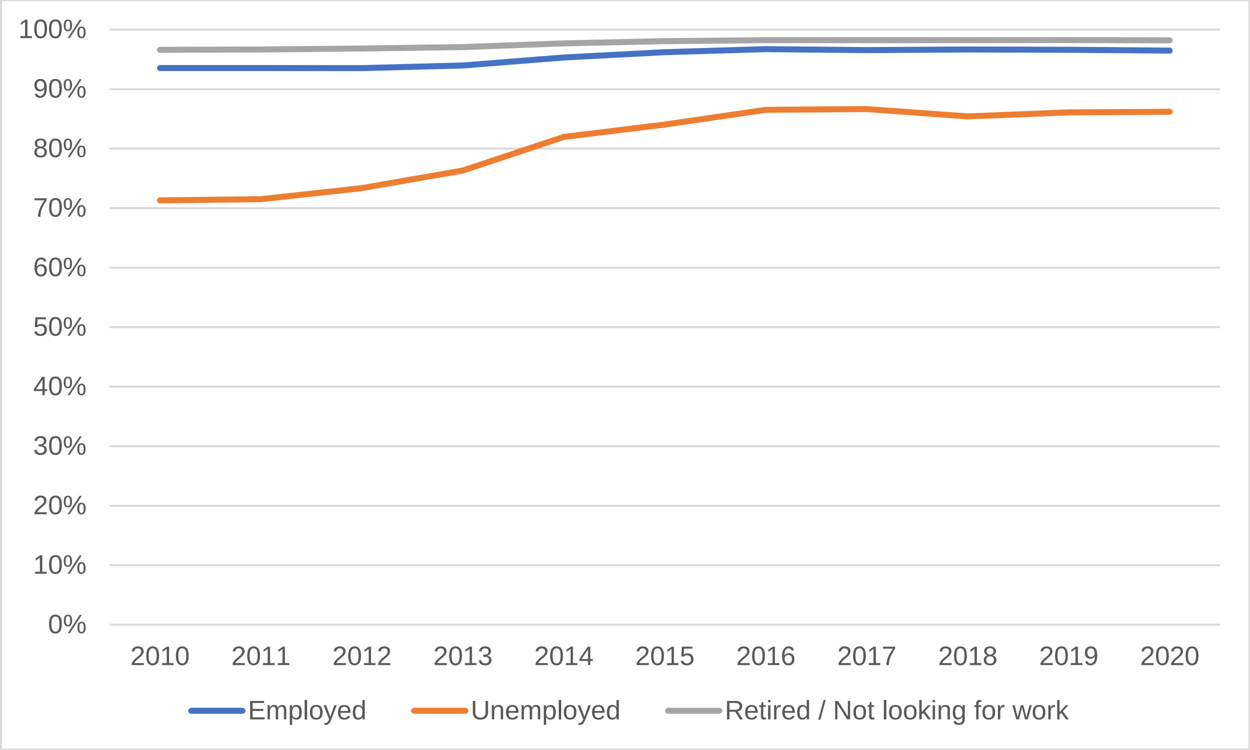

Notes: ACS is American Community Survey. ACS data on Veteran Employment Status was weighted to be nationally representative.

© 2024 Wagner TH et al. *JAMA Network Open.*

eFigure 9: Share of Veterans Reporting Any Insurance Coverage by Employment Status (BRFSS)

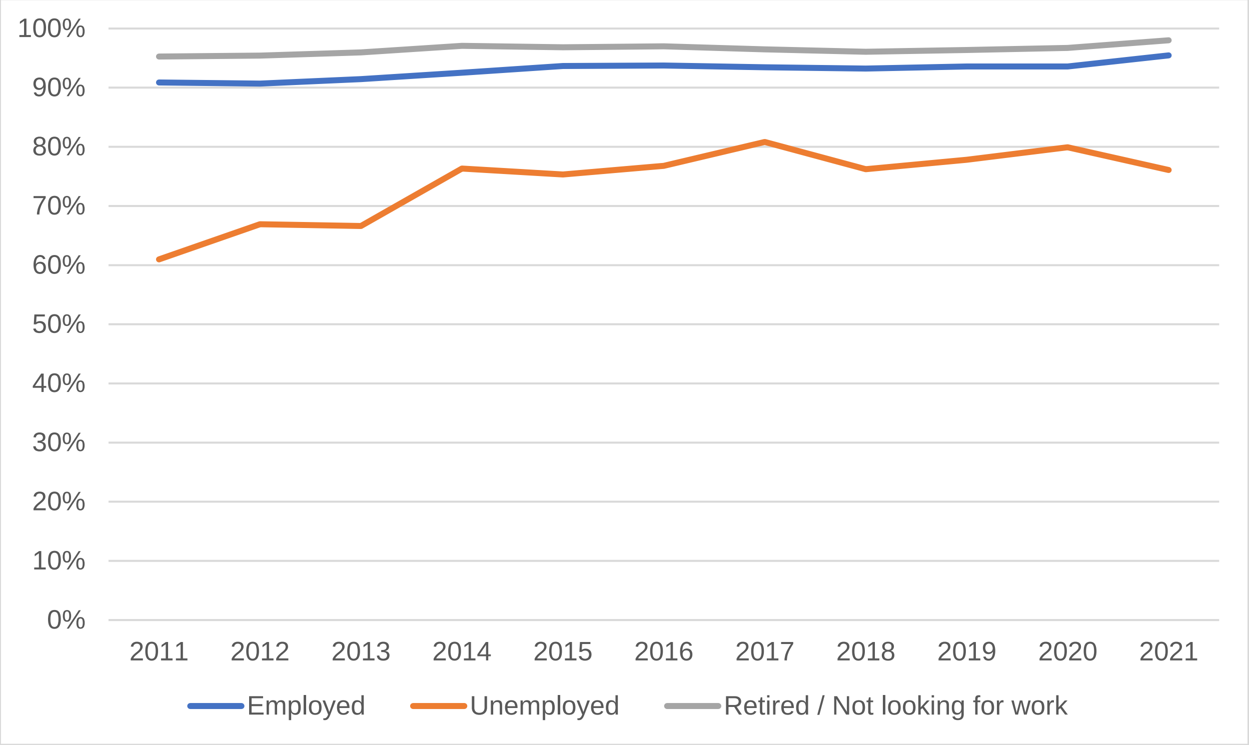

Notes: BRFSS is Behavioral Risk Factor Surveillance Survey. BRFSS data on Veteran Employment Status was weighted to be nationally representative at the state level.

© 2024 Wagner TH et al. *JAMA Network Open.*

eFigure 10: Share of Veterans Reporting Any Insurance Coverage by Employment Status (NHIS)

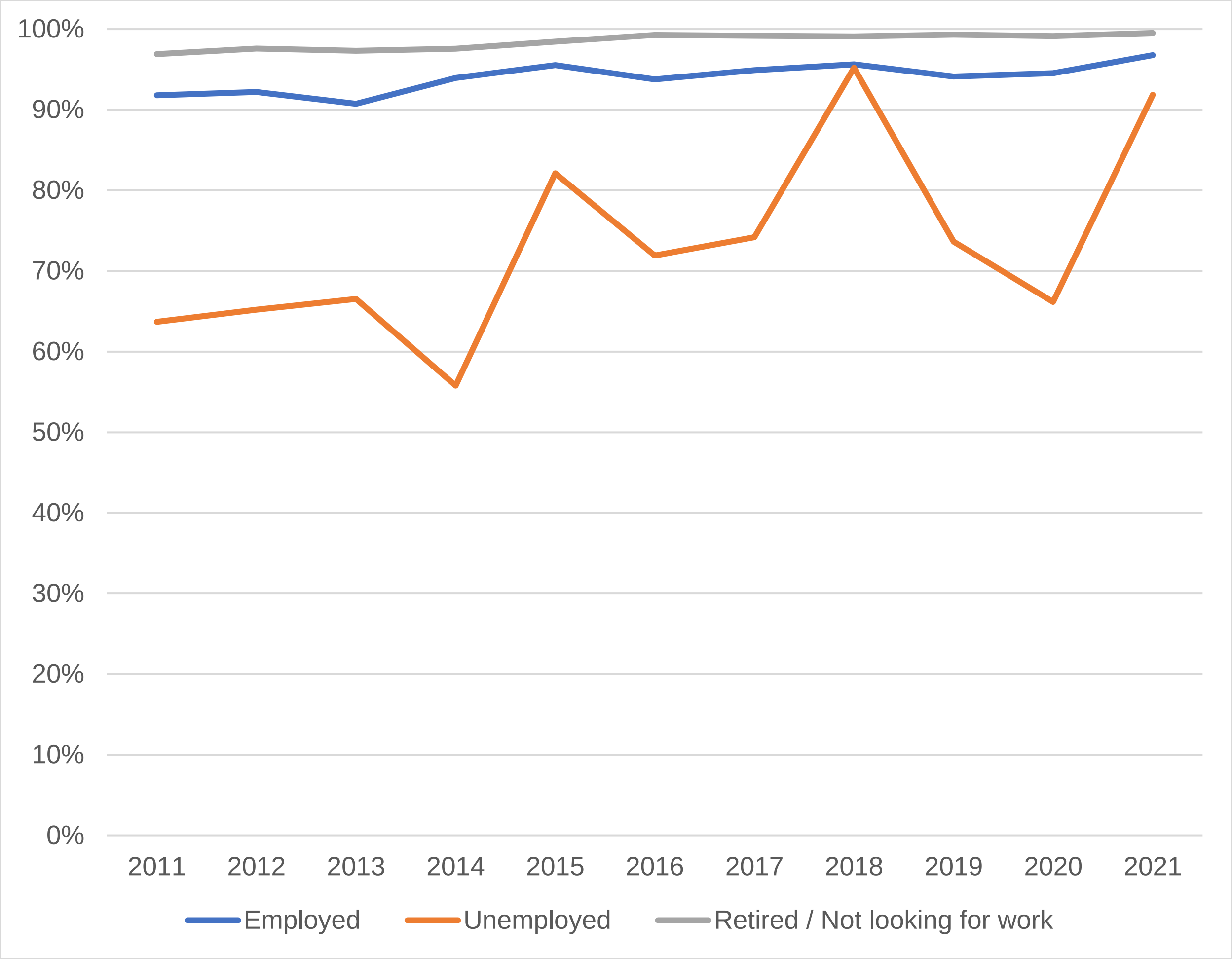

Notes: NHIS is National Health Interview Survey. NHIS data on Veteran employment status was weighted to be nationally representative.

© 2024 Wagner TH et al. *JAMA Network Open.*

eTable 2: Veteran Insurance Coverage by Race and Ethnicity (ACS)

	ACS		NHIS	
	**Non-Hispanic ethnicity**	**Hispanic ethnicity**	**Non-Hispanic ethnicity**	**Hispanic ethnicity**
		Any Insurance		
white	96%	94%	96%	94%
Black	94%	93%	94%	93%
Asian	96%	95%	95%	99%
Other	93%	93%	91%	92%
		VA enrolled		
white	30%	30%	19%	24%
Black	35%	29%	25%	26%
Asian	25%	30%	16%	14%
Other	35%	29%	31%	33%
		Private insurance		
white	64%	56%	56%	49%
Black	54%	49%	48%	51%
Asian	61%	56%	53%	68%
Other	52%	52%	43%	47%
		Medicare		
white	52%	29%	53%	32%
Black	30%	16%	30%	9%
Asian	31%	23%	36%	14%
Other	34%	23%	40%	28%
		Medicare Advantage		
white	--	--	12%	8%
Black	--	--	6%	3%
Asian	--	--	10%	11%
Other	--	--	9%	3%
		Tricare		
white	19%	26%	14%	17%
Black	26%	37%	19%	13%
Asian	33%	37%	23%	15%
Other	25%	27%	20%	17%
		Medicaid		
white	8%	9%	3%	5%
Black	13%	12%	7%	11%
Asian	8%	9%	4%	10%
Other	13%	11%	7%	8%

© 2024 Wagner TH et al. *JAMA Network Open.*

Notes: ACS is American Community Survey. NHIS is National Health Interview Survey. Weighted to be nationally representative. NHIS data for 2011-2021. ACS data for 2010-2020

© 2024 Wagner TH et al. *JAMA Network Open.*

eFigure 11. Insurance Coverage Rates for Veterans by Race (ACS)

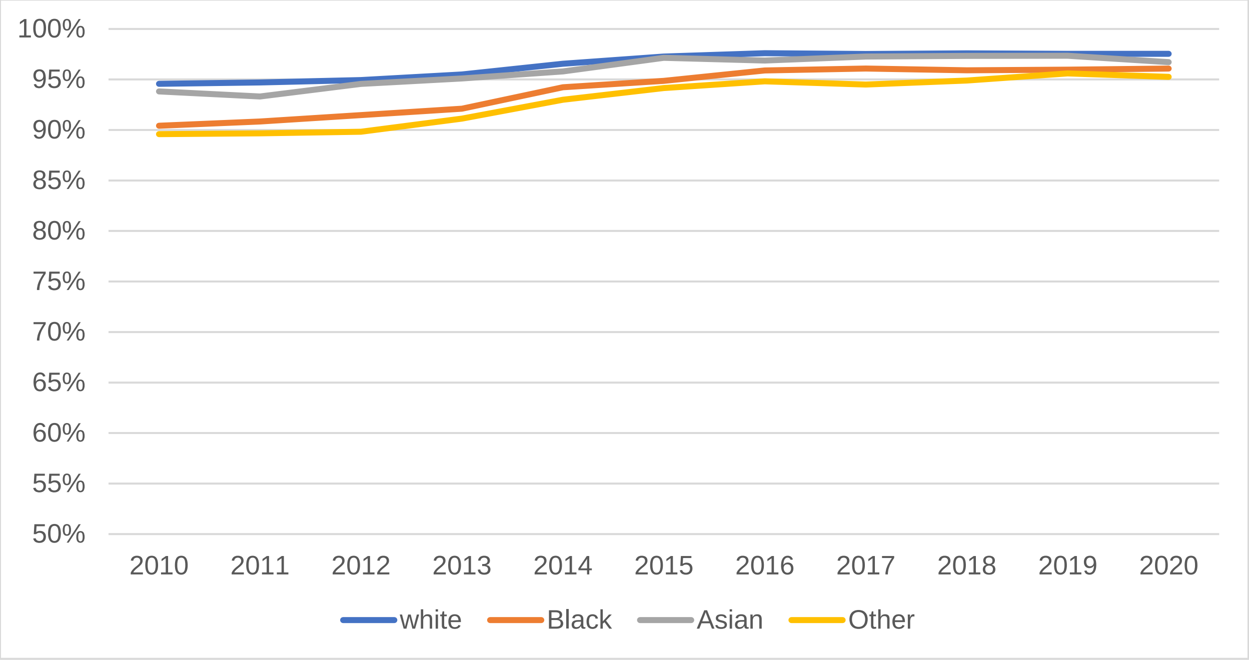

Notes: ACS is American Community Survey. ACS data on Veteran insurance coverage by race was weighted to be nationally representative.

© 2024 Wagner TH et al. *JAMA Network Open.*

eFigure 12. Insurance Coverage Rates for Veterans by Race (BRFSS)

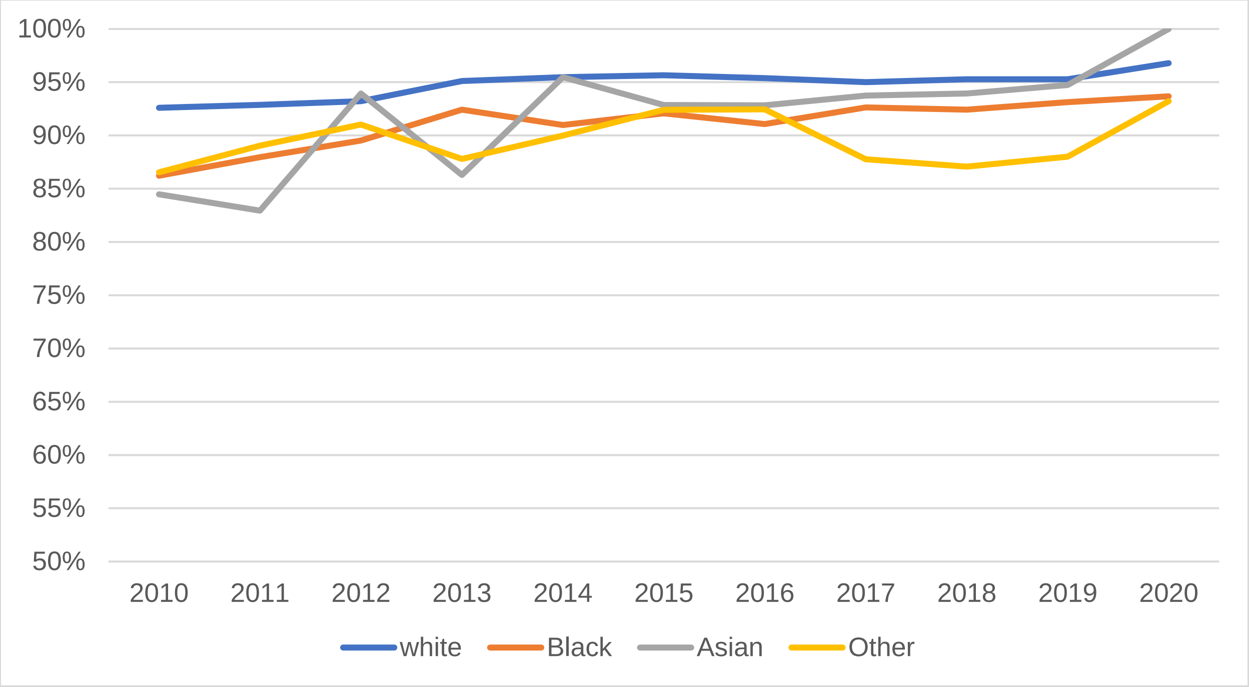

Notes: BRFSS is Behavioral Risk Factor Surveillance Survey. BRFSS data on Veteran insurance coverage by race was weighted to be nationally representative at the state level.

© 2024 Wagner TH et al. *JAMA Network Open.*

eFigure 13. Insurance Coverage Rates for Veterans by Race (NHIS)

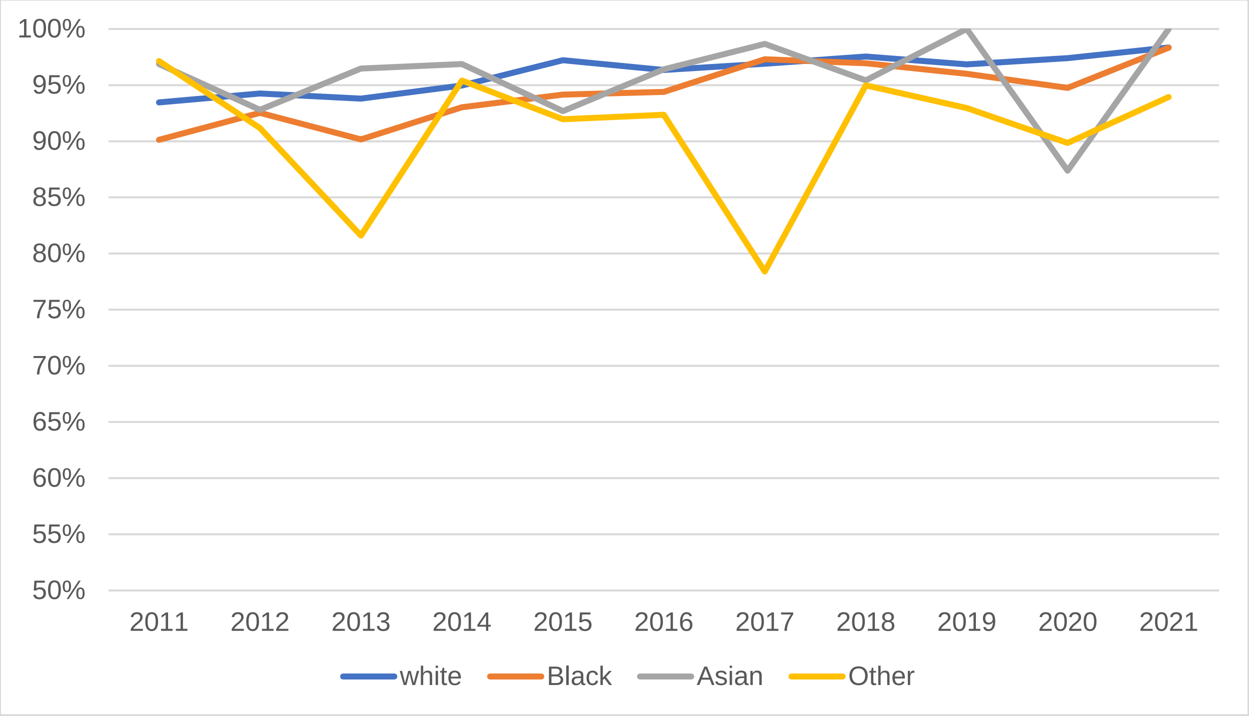

Notes: NHIS is National Health Interview Survey. NHIS data on Veteran insurance coverage by race was weighted to be nationally representative.

© 2024 Wagner TH et al. *JAMA Network Open*.

eTable 3: Veteran Insurance Coverage by Age

	18-24	25-34	35-44	45-54	55-64	65+
			**NHIS**			
2011	75%	79%	87%	90%	92%	99%
2012	85%	86%	87%	90%	91%	100%
2013	83%	80%	88%	86%	93%	100%
2014	80%	85%	90%	90%	93%	100%
2015	91%	89%	91%	95%	96%	100%
2016	85%	87%	90%	94%	95%	100%
2017	78%	93%	93%	95%	94%	100%
2018	98%	95%	94%	95%	95%	100%
2019	86%	92%	95%	94%	94%	100%
2020	64%	92%	95%	94%	95%	100%
2021	92%	98%	98%	97%	95%	100%
			**ACS**			
2010	88%	86%	88%	88%	93%	100%
2011	90%	87%	89%	88%	93%	100%
2012	91%	87%	89%	89%	93%	100%
2013	93%	88%	90%	90%	93%	100%
2014	94%	91%	92%	92%	95%	100%
2015	95%	93%	94%	94%	95%	100%
2016	97%	94%	95%	95%	96%	100%
2017	97%	94%	95%	95%	95%	100%
2018	97%	94%	95%	95%	95%	100%
2019	96%	94%	95%	95%	95%	100%
2020	96%	94%	94%	95%	95%	100%
			**BRFSS**			
2010	88%	86%	88%	88%	93%	100%
2011	83%	83%	88%	88%	91%	99%
2012	86%	86%	88%	89%	91%	99%
2013	88%	84%	89%	87%	92%	99%
2014	89%	87%	91%	91%	94%	99%
2015	91%	90%	92%	94%	95%	99%
2016	92%	91%	92%	94%	94%	99%
2017	91%	92%	93%	94%	94%	99%
2018	92%	92%	94%	94%	94%	99%
2019	92%	91%	94%	94%	94%	99%
2020	89%	92%	94%	94%	94%	99%
2021	91%	98%	98%	96%	95%	100%

© 2024 Wagner TH et al. *JAMA Network Open.*

Notes: NHIS is National Health Interview Survey. NHIS survey results were weighted to be nationally representative. ACS is American Community Survey. ACS survey results were weighted to be nationally representative. BRFSS is Behavioral Risk Factor Surveillance Survey. BRFSS survey results were weighted to be nationally representative at the state level.

© 2024 Wagner TH et al. *JAMA Network Open*.
