# Supplementary material for: Health Insurance Enrollment Among US Veterans, 2010-2021
Source: JAMA Netw Open. Author manuscript; Available in PMC 2024 Oct 28. (PMC11514437; doi:10.1001/jamanetworkopen.2024.30205)
Supplement: Supplement 2 — Data Sharing Statement [file NIHMS2026864-supplement-Supplement_2.pdf]

Data Sharing Statement

Wagner. Health Insurance Enrollment Among US Veterans, 2010-2021. *JAMA Netw Open*. Published August 26, 2024. doi:10.1001/jamanetworkopen.2024.30205

Data
Data available: No

Additional Information
Explanation for why data not available: Data for the National Health Interview Survey (NHIS), American Community Survey (ACS), and Behavioral Risk Factor Surveillance System (BRFSS) are available to the public. However, the Department of Veterans Affairs Survey of Enrollees (SOE) is restricted to researchers within the VA with a secondary request process.
